# Editorial: Pharmacokinetic differences of drugs and their regulatory mechanisms under dual status including normal and diseased organism

**DOI:** 10.3389/fphar.2022.1063434

**Published:** 2022-12-12

**Authors:** Zipeng Gong, Jie Zhou, Ling Ye, Guo Ma, Yanfang Xian, Kaustubh Kulkarni

**Affiliations:** ^1^ State Key Laboratory of Functions and Applications of Medicinal Plants, Guizhou Provincial Key Laboratory of Pharmaceutics, School of Pharmacy, Guizhou Medical University, Guiyang, China; ^2^ Key Laboratory of Basic Pharmacology of Ministry of Education, Zunyi Medical University, Zunyi, China; ^3^ Guizhou Provincial Engineering Research Center for the Development and Application of Ethnic Medicine and TCM, Guizhou Medical University, Guiyang, China; ^4^ NMPA Key Laboratory for Research and Evaluation of Drug Metabolism, Guangdong Provincial Key Laboratory of New Drug Screening, School of Pharmaceutical Sciences, Southern Medical University, Guangzhou, China; ^5^ School of Pharmacy, Fudan University, Shanghai, China; ^6^ School of Chinese Medicine, Faculty of Medicine, The Chinese University of Hong Kong, Hong Kong Special Administrative Region, Hong Kong, Hong Kong SAR, China; ^7^ Boundless Bio Inc., San Diego, CA, United States

**Keywords:** dual status, pharmacokinetic differences, drug metabolizing enzymes, transporters, nuclear receptors, pharmacokinetic-pharmacodynamic model

Dual status includes healthy state and pathological state. Pharmacokinetics (PKs) is a quantitative study of drug dynamic changes including the process of absorption, distribution, metabolism and excretion (ADME). In the course of innovative drug development, PK study is as important as pharmacodynamic and toxicological research in evaluating new drugs, which also plays a vital role in reducing the risks of new drug development existing in preclinical and clinical study. However, it is irrational that the current preclinical PK data largely come from healthy animals. Firstly, given that drugs are largely taken by patients, they are the ultimate consumers of drugs. Secondly, pathological body could lead to the change of drug metabolizing enzymes, drug transporters and intestinal microflora, which bring about the change of ADME of drugs and have adverse effect of the use of drugs in clinic. Therefore, carrying out the PK studies under pathological conditions would be more significant and closely related to clinic (Gong, et al., 2015).

Generally speaking, compared with the healthy state, the function of some tissues and organs of the body in the pathological conditions might be harmed. For instance, the expression and activity of drug metabolizing enzymes (DMEs) under diabetic conditions were altered (Chen et al., 2018). When the body was under rheumatoid arthritis conditions, the kind and amount of intestinal microflora were changed (Tajik et al., 2020). Moreover, the gap between adjacent intestinal cells in patients with irritable bowel syndrome was increased (Gong et al.). It is worth mentioning that these changes would alter the PK behavior of the drug. Also, inflammation could regulate the change of DMEs and transporters, and its possible mechanisms might largely involve inflammation-related signaling pathways and nuclear receptors, etc. (Wu et al., 2019), which led to changes in the PK process, efficacy and toxicity of drug. Therefore, the research on regulatory mechanisms causing the PK differences of drugs between healthy state and pathological state should be paid more attentions.

In the main frame of this Research Topic, 16 contributions have been published including but not limited to the following subjects: comparative PKs between healthy state and pathological state, the changes in the expression and activity of DMEs and transporters in pathological state, ADME/toxicity of drug and their regulatory mechanisms, drug-drug interactions mediated by nuclear receptors, DMEs and transporters based on the way of PKs, transcriptomics and metabolomics.

Wang et al. found that the Huangqi Liuyi decoction extract could be effective for improving the renal function when treating diabetic nephropathy. Moreover, the tissue distribution of six active ingredients in Huangqi Liuyi decoction extract could be affected by diabetic nephropathy state.

Zhang et al. compared PK of four main active ingredients of Liandan Xiaoyan Formula(LXF) in healthy and ulcerative colitis rats. They found that main active ingredients of LXF in the ulcerative colitis group had higher exposure than in the healthy group.

Liu et al. investigated the PK differences of three active diterpenoids of *Rabdosia serra* extract in healthy and concanavalin A-induced liver injury rats. They found that the PK process of three active diterpenoids in *Rabdosia serra* extract was influenced by liver injury.

Chen et al. found the compatibility of fuzi and ginseng could improve markedly the *in vivo* exposure of five bioactive ingredients including mesaconitine, benzoylaconitine, benzoylmesaconitine, benzoylhypaconitine, and songorine.

Chen et al. studied the PK behavior and main metabolites of anwulignan in mice. They identified that anwulignan might undergo the enterohepatic circulation. Moreover, seven metabolites were also identified, which mainly involved the demethylation, hydroxylation, dehydroxylation, and demethoxylation.

Liu et al. found *Rehmanniae Radix* (RR) displayed an inspiring antidiabetic effect by reducing the fasting blood glucose and insulin resistance, upregulating the mRNA and protein expressions of transient receptor potential vanilloid 1 (TRPV1), and downregulating mRNA expression of stearoyl-CoA desaturase 1 (SCD1). Therefore, induction of TRPV1 and inhibition of SCD1 by RR was possibly one of its antidiabetic mechanisms.

Ruan et al. evaluated an untargeted metabolomic strategy for discovering the biomarker of breast cancer, which would lay a base for discovering biomarker and investigating the disease mechanism.

Xu et al. elucidated the mechanism of Huang Qin decoction for treating the diabetic liver injury by the combination of metabolomics and network pharmacology.

Ke et al. investigated the mechanism of action of geniposide treating rheumatoid arthritis (RA) by employing metabonomic analysis of abnormal sphingolipid metabolism in RA synovial fibroblasts in hypoxia microenvironment and intervention of geniposide.

Chen et al. established a population pharmacokinetic (PPK) model for isoniazid (INH) and its major metabolite acetylisoniazid (AcINH) in healthy Chinese participants and tuberculosis patients and assessed the role of the NAT2 genotype on the transformation of INH to AcINH.

Wang et al. found JBP485 could save from imipenem nephrotoxicity in rabbits and human kidney 2 cells by increasing imipenem stability and reducing its intracellular accumulation by simultaneously inhibiting the renal organic anion transporters and dehydropeptidase-I.

Zhang et al. demonstrated that midazolam could ameliorate CCl_4_-induced acute liver injury and oxidative stress *via* activating the nuclear-factor erythroid 2-related factor 2 (Nrf2) signaling pathway.

Wang et al. evaluated the interaction of danshen and rivaroxaban by using the liver microsomes of rat and human. They found Danshen tablet could inhibit the metabolism of rivaroxaban due to its lipid-soluble ingredients such as dihydrotanshinone I could strongly inhibit the activity of CYP3A and CYP2J.

Xie et al. summarized the solubility, permeability, molecular structure and molecular weight characteristics of various natural medicines that affected cerebral metabolism (NMCs), and reviewed drug delivery systems that enhanced the PK and pharmacodynamic features of NMCs. Moreover, the structure-based *in vivo* metabolic reactions regulated by DMEs and metabolites of NMCs were also reviewed.

Yu et al. identified new platelet-derived growth factor receptor α (PDGFRA) inhibitors from traditional Chinese medicine (TCM) *in silico* and verified their effects of targeting PDGFRA and radioiodine uptake, which could provide a potential drug lead for developing a new radioiodine-refractory thyroid cancer therapy.

Li et al. summarized the research progress of the material composition, pharmaceutical production, clinical application and pharmacology mechanism of various TCM film agents, which might provide a comprehensive reference for further development and utilization of TCM film agents.

Therefore, given that drugs are largely taken by patients, the current topic focused mainly on PK differences of drugs and their regulatory mechanisms under healthy state and pathological state, which could provide reference and ideas for individualized safety and effective drug use, and optimizing the drug evaluation system based on disease states.
